# Airborne signals and abiotic factors: the neglected side of the plant communication

**DOI:** 10.1080/19420889.2020.1767482

**Published:** 2020-05-18

**Authors:** Marco Landi

**Affiliations:** a Department of Agriculture, Food and Environment, University of Pisa, Pisa, Italy; b CIRSEC, Centre for Climatic Change Impact, University of Pisa, Pisa, Italy

**Keywords:** Abiotic stress, environmentally controlled conditions, global change factors, infochemicals, inter-plant communication, intraspecific, volatile organic compounds, volatilome

## Abstract

A relevant number of reports have examined the role of airborne signals in plant-plant communication, indicating that volatile organic compounds (VOCs) can prime neighboring plants against pathogen and/or herbivore attacks. Conversely, there is very limited information available on the possibility of the emission of VOCs by emitter plants under abiotic stress conditions, which may alert neighboring unstressed plants and prime these individuals (receivers) against the same stressors. The present opinion paper briefly reviews a few reports examining the effect of infochemicals produced by emitters on receiver plants subjected to abiotic stresses typical of global climate change. The ecological implications of these dynamics, as well as some concerns related to the potential roles of inter-plant communication in environmentally controlled experiments, have arisen. Some possible inter-plant communications applications (biomonitoring and biostimulation), mediated by airborne signals, and some directions for future studies on this topic, are also provided.

## Introduction

There was a presumptuously anthropocentric assumption that communication is only possible in the presence of a nervous system that plants lack, thus considered deaf and incapable of communication until the mid-1900s [1]. In the 1960s, Cleave Backster was the first who put forward the idea that plants have the capacity to receive and transmit messages [2], which is exactly what we usually refer to with the term “communication.

Before conducting experiments on plants, Dr. Backster (1924–2013) was a polygraph examiner with the CIA. His studies were inspired by the work of the physicist Jagadish Chandra Bose (1858–1937), who observed for the first time the ability of plants to sense the environment. Bose invented various devices and instruments to measure the electrical signals in plants, of which “crescograph”, the most famous, was used to measure plant responses to various stimuli. Based on the analysis of the variation of the cell membrane potential of plants under different circumstances, he hypothesized that plants and their different organs were sensitive, exhibiting electric responses under mechanical or other stimuli, which is indicative of plant/organ excitation. Backster moved ahead with his studies and not only proposed the idea that plants are able to perceive environmental stimuli but also hypothesized that they can exchange information with other plants, thus posing the bases for explaining plant-plant communication. The scientific community expressed skepticism about Backster’s data interpretation [3] for different reasons, including the following: i) he did not follow a scientific approach in his experiments; ii) the reliability of the polygraph test was and has still been questioned; iii) replication of Backster’s experiments under controlled conditions failed to achieve similar results in many cases [4], which all were rebutted by different scientific explanations [5]. Even though his theories were not supported scientifically, his great achievement was to draw the scientific community’s attention to the possibility that plants respond to external stimuli and interact with the environment and other plants, suggesting that plants are not just passive entities subjected to environmental conditions and attack by other organisms or accumulate biomass for animal feed.

The previous works marking the first milestone for “talking trees” [6], as well as other experimental reports, examining airborne signals between plants [7,8]. [6], particularly, suggested for the first time the plant-plant communication triggered by airborne signals. The hypothesis was based on the observation that damaged sugar maple seedlings responded in a manner similar to that of the nearby undamaged maples, which led to increased levels of phenolics and hydrolyzable and condensed tannins. The authors suggested that an airborne cue originating in damaged tissues may stimulate biochemical changes in neighboring plants that could influence the feeding and growth of phytophagous insects. Even though the interpretation of the results was criticized [9,10], from that time onward, it has been widely accepted that VOCs can serve as infochemicals in the communication between plants [1,11].

To date, we still know very little about the plant-environment interactions mediated by VOCs, and most underlying physiological, biochemical, and genetic mechanisms remain to be discovered; however, several studies have demonstrated that plants growing in nature can benefit from a VOC-mediated communication [1]. Now it is obvious that plants are as sophisticated in behavior as animals, but, besides, they have great adaptive potential due to their sessility. The plants’ inability to escape environmental stressors brings out extraordinary plasticity to perceive and respond to fluctuating or drastic environmental cues [12]. However, human activities and population growth pose serious threats to the ecological dynamics, including plant-to-plant communication, given that the increase in the amplitude of global change factors occurs faster than the adaptation ability of plants to such changes.

The aim of the present opinion paper is to draw attention to the need for more research on plant-plant communication triggered by stress-induced VOCs to acquire basic knowledge of ecology and dynamics of plant communities, evaluate possible interferences related to the release of VOCs in environmentally controlled experiments, study the language of VOCs in plants under abiotic stress for environmental monitoring, and exploit VOCs that promote abiotic stress tolerance to stimulate the biosynthesis of target metabolites in receivers.

## Airborne plant-plant communication triggered by abiotic factors

Plants are extremely efficient not only in using CO_2_ (only present in the air at below 400 ppm) to build tens-meter-high trees but also in investing a huge amount of freshly assimilated carbon into volatile organic compounds (VOCs), which are released back into the atmosphere [13]. For example, trees annually produce 500 Gt of isoprene along with several other classes of monoterpenes, sesquiterpenes, and other VOCs [14]. It is known that VOC emission is not a mere waste of assimilated carbon, but the release of VOCs into the environment regulates key ecological functions such as attracting pollinators, scavenging of reactive oxygen species, inducing plant defense against herbivores and pathogens [13,15–17], and mediating intraspecific inter-plant communication [16].

The term “communication” often has different meanings; it is conceivable that information dissemination is the minimum requirement, regardless of the intention to communicate or the benefits for both emitter and receiver. For the sack of the truth, some researchers reported that an interaction in which plants increase the fitness of their neighbors without improving their own fitness results in an evolutionary disadvantage for the emitter, and therefore represents ‘eavesdropping’ rather than true communication [1]. Therefore, it is still under debate and beyond the scope of the present mini-review whether the infochemicals evolve in parallel with the “voluntariness” by the emitter to transmit a message for the benefit of the neighboring plant belonging to the same species, or with the capability of neighboring plants to “eavesdrop” on VOCs from emitter plants.

A considerable amount of literature exists on plant-plant communication triggered by biotic factors. For example, it has been extensively proven that VOCs emitted by plants that are subjected to attacks by herbivores or pathogens can elicit responses in neighboring plants (not under attack), alerting receivers in case of future attacks [19]. It has also been demonstrated that herbivory damage may lead to the newly induced VOCs emitted by plants [20] or stimulation of the biosynthesis of some main constitutively-emitted VOCs [21].

In contrast, there is only a little information available on airborne-mediated inter-plant communication triggered by abiotic factors, and only a few investigations have been conducted [22–26], which contrast sharply with the necessity to understand how plants are able to build awareness of abiotic hazards, especially in the era of global change [17]. Plants are almost continuously exposed to a large variety of abiotic stress factors including the diurnal variation of temperature, irradiance, UV, drought events, flooding (caused by heavy rain), salinity, and metal toxicity, and exponential increases in their frequency and amplitude have been observed in some areas due to global change. All these factors alter the fingerprint of infochemicals emitted by plants [15,17,27,28], thereby altering plant-environment interactions mediated by VOC emission [17,29,30].

Concerning the condition of reduced water availability, the biotic-abiotic interactions, mediated by infochemicals, in which drought-stressed plants induce VOC emission in unstressed receivers, which increases the attraction of parasitic wasps, were described [24]. This mechanism was hypothesized to improve protection against aphids under conditions of low water availability.

Metals are now widespread environmental pollutants, and their toxicity is an increasing concern for ecological, nutritional, and environmental reasons. These pollutants ultimately released by an increasing number of diverse anthropogenic sources (industrial effluents and wastes, urban runoff, sewage treatment plants, boating activities, fungicide in agricultural runoff, domestic garbage, dumps, and mining operations), have progressively affected different ecosystems [31]. Changes in the VOC profile, attributable to exposure of the plant to excess concentrations of metals, have been recently demonstrated with an excess of Zn by [22]. The authors observed that Zn treatment increased VOC emission without changing the volatilome profile. In particular, the authors reported the accumulation of stress priming, such as some C_6_-aldehydes and terpenes, which were involved in plant-plant communication [29,32]. These preliminary results are, therefore, supportive of possible changes in plant-plant communication under conditions of excess edaphic metal ions.

Under salinity stress, another common problem caused by global change, [26] found that *Arabidopsis* receivers resulted more tolerant than emitters, as showed by higher seed germination and increased plant growth [26], suggesting that salt-promoted changes in VOCs are relevant in priming salt tolerance in neighboring [unstressed) plants. [23] observed that the receiver plants of *Vicia faba* maintained a higher photosynthetic rate, photosystem II efficiency, and relative growth rate than emitters when subjected to the same level of NaCl, highlighting that leaf volatiles can act as airborne signals in salt stress communication. Recently, [25] demonstrated that emitter sweet basil plants subjected to salt stress induced significant alterations in volatilome and metabolomic profiles of (untreated) receivers. When receivers were subsequently subjected to salt stress, early plant senescence was observed, which was related to the higher capacity of plants to produce vital seeds under salinity stress. These results reveal that priming by emitters does not offer a clear advantage to the physiological performance of receivers [23,26], but increases their reproductive success. The authors correlated this phenomenon to the capability of receivers to perceive infochemicals from the emitter and accordingly modulate their metabolism as described by the leaf metabolomics analyses.

In contrast to the results of the study conducted by [1], previous findings of [25] seem supportive of the possibility of “true communication” as it brings an advantage to the emitter/receiver species rather than to individual emitter or receiver, given that neither the emitter nor the receiver gained a distinct advantage from VOC-induced priming [see the previous section for the details).

Besides the new mechanism described in [25], the following questions still remain open: “Are receivers able to propagate the airborne-triggered signal? And are receivers better or worse than emitters in doing that?”, “Is the higher level of seed production of receivers only related to the effects of VOCs released by emitters on plant metabolism? Or this is (also) attributable to changes in the VOC profile of receivers and consequently in VOC-mediated interaction between receiver plants and pollinators?”. The authors also conclude that the neglected possibility of plant communications under abiotic stress should be seriously considered in environmentally controlled experiments. Indeed, under abiotic stress conditions, plants subjected to different treatments might be reciprocally influenced by their VOC emission when not physically separated by any barriers for preventing diffusion of VOC. Some aspects related to the latter point will be briefly examined in the next paragraph, along with limits, directions, and opportunity to study plant communication triggered by abiotic stress-induced VOCs.

## Awareness, concerns, and possibilities about plant-plant communication by VOCs

### VOC interference in environmentally controlled experiments

The idea that airborne signals between plants subjected to different treatments may affect plant performance in environmentally controlled experiments may be shocking to most readers since the majority of experiments in plant physiology are carried out in the controlled environment to avoid the unwanted effect of external factors. To date, not too much consideration has been given to the communication among plants subjected to different treatments through airborne signals. But if this possibility emerges clearly from the experimental trials aimed at describing the mechanisms related to VOC-mediated plant-plant communication (see the previous section), why this possible influence of emitter infochemicals to receiver performances has never been postulated before in other kinds of experiments under controlled conditions without dealing with VOC-mediated communication?

**Figure 1.**
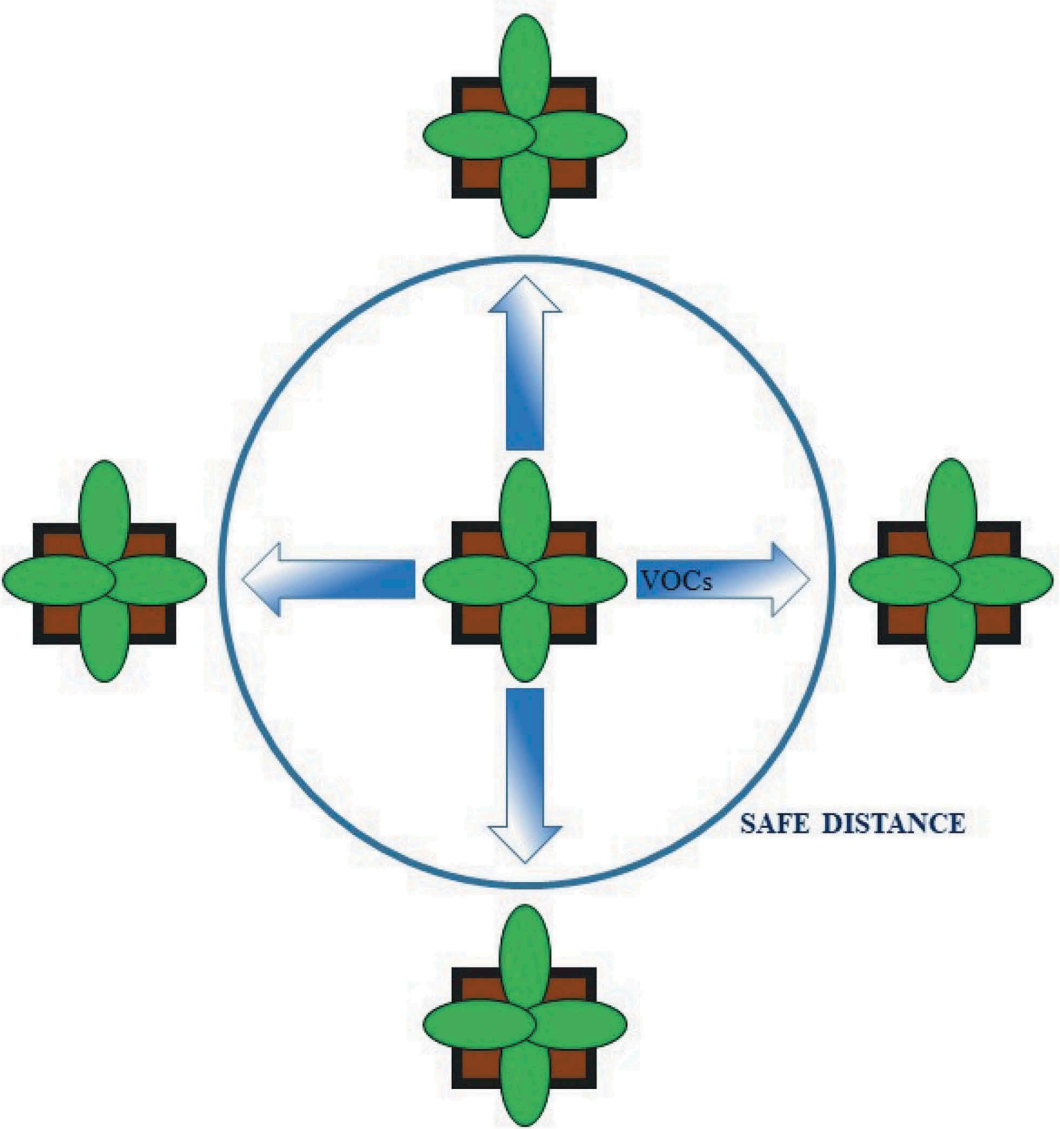
A schematic representation of release and diffusion of volatile organic compounds (VOCs), and maintaining a “safe distance” in environmentally controlled experiments.

**Figure 2.**
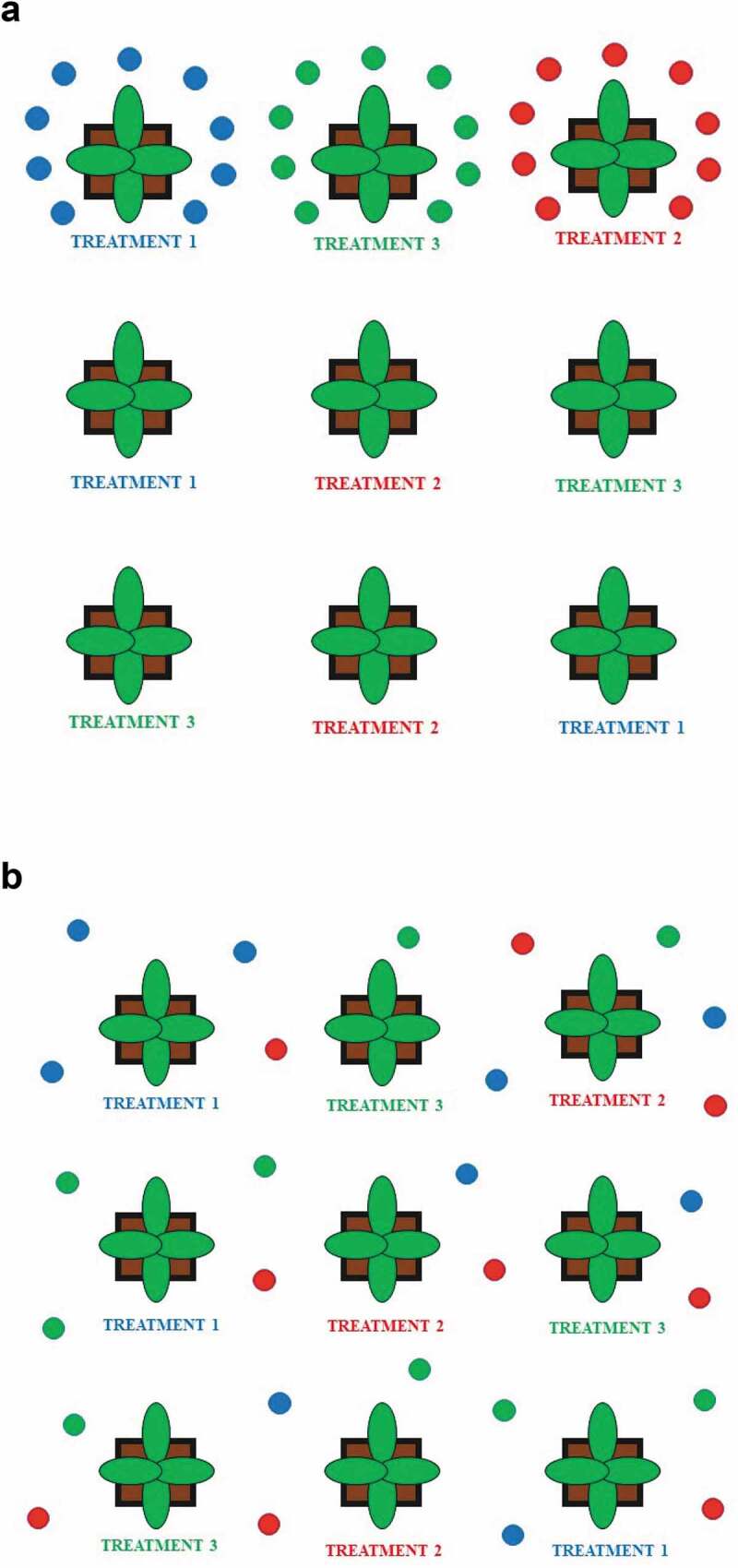
(a) A schematization of different volatile organic compounds (VOCs) released by plants exposed to three different treatments (represented by blue, red, and green spots); (b) Diffusion of different VOCs in the environment, and the production of the VOC blend in environmentally controlled experiments.

It is arguable that experimental results have never reported such influence (at least in published works); for example, a degree of variability, greater than the common biological variability, in physiological and biochemical parameters of plants, subjected to the same treatments, but positioned at different distances from the plants exposed to other treatments, has never been pointed out. In this case, two factors have to be considered:

- **The distance between plants**: volatile diffusion in controlled environments might be limited by reduced airflow (especially when no ventilation is provided), and a certain distance between plants is enough to avoid a possible mutual influence of VOCs. However, future research should consider the possibility of plant responses in a gradient-dependent way to VOC emission from neighboring plants to determine the “safe distance” between plants (or groups of plants) exposed to different treatments (Figure 1).
- **The experimental design**: the application of a completely-randomized experimental design makes it possible to mix the VOCs emitted by plants subjected to different treatments, thereby changing the VOC bouquet perceived by all plants. In this case, this possible unwanted effect can be evaluated by comparing a completely randomized block design with randomized experiments conducted using plants at a “safe distance” from neighboring plants (Figure 2).

### VOC signals, plant communication, and human applications: limits and perspectives

The interest in understanding plant communication has developed constantly over the last five years, as shown by the increasing number of publications on this topic. Understanding of VOC-mediated communication between plants can be particularly important for describing intraspecific inter-plant ecological interactions and understanding how abiotic stress changes these dynamics, exploiting VOCs in plant-plant communications regulated by abiotic factors to use some model plant species as bioindicators, and exploiting elicited VOC to stimulate the biosynthesis of targeted metabolites in receiver plants, thereby developing a “clean” and bioactive-enriched product. However, understanding of plant responses triggered by infochemicals, which are emitted by neighboring stressed plants, is extremely difficult for different reasons.

First, plants emit a wide array of volatile compounds, which are, in most cases, species-specific and age-dependent [33]. Therefore, it is very difficult to find consistent cause-effect relations developed by one or a few volatiles in different species. Second, in some cases, plants respond to abiotic stress factors by changing their volatile profiles [25], while in other cases, they only produce larger amounts of some key VOCs [22]. In addition, plants can be naturally subjected to multiple stressors, and plant responses to stress combinations cannot always be extrapolated from responses to individual stress factors; multiple stress factors could have additive, neutral, or opposing effects on VOC emission [33].

**Figure 3.**
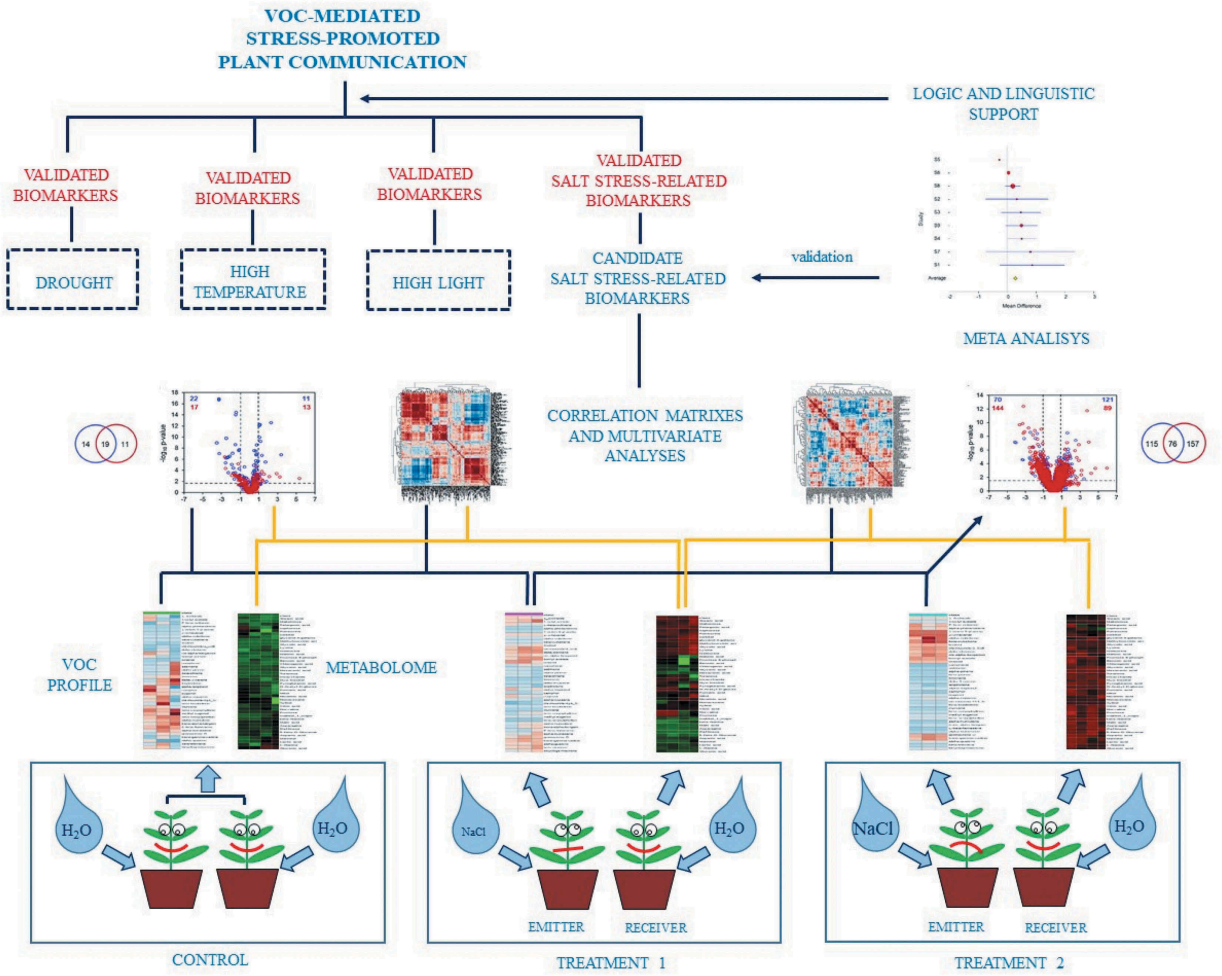
An experimental approach to describe volatile organic compound (VOC)-mediated plant communication under abiotic stress conditions. The correlation between the volatilome of emitter plants and the metabolome in receiver plants allows describing possible biochemical markers of particular abiotic stress. By imposing different abiotic stresses on the same species, it would be possible to obtain stress-specific quantitative markers from the association of changes in emitter’s VOCs and receiver’s metabolites. The interaction of different abiotic stress factors could be used to develop an integrated model of plant communication.

“*How can this system become more informative*?” To date, there are a very limited number of reports on changes in the VOC profile of emitter plants subjected to abiotic stress and the responses of receivers at the physiological level [23,24]. This represents a starting point to figure out a possible effect of VOCs from emitters on performances of the receivers, but to strengthen this relationship, it is necessary to associate the analysis of the plant volatilome with a deep metabolomics investigation. Then, it is possible to establish the most representative VOC blends of emitters and the metabolic changes in receiver plants by multivariate analyses [25]. If this approach is applicable to experiments, which attempt to establish a cause-effect relationship between different levels of the single abiotic stresses, it is possible to find some quantitative markers related to the imposed stress level for the species under investigation (Figure 3). Indeed, to date, there is little evidence that plants have a dedicated “olfactory” system similar to the one in animals, and, at present, a VOC receptor has only been described for ethylene [34]. Other physicochemical (changes in membrane gradient) and metabolic processes (reaction of emitter’s VOCs with receiver’s organic compounds) could be implicated in VOC detection and response [35].

The aforementioned approach is essential to draw attention to key putative biomarkers of selected stress factors. Thereafter, the analysis of candidate volatile/metabolomic biomarkers should be validated with meta-analyses of datasets related to the same stress factor from the existing literature to obtain a few biomarkers used as quantitative traits for the future investigations (Figure 3). By imposing different abiotic stresses on the same species, it would be possible to obtain stress-specific quantitative markers from the association of changes in emitter’s VOCs and receiver’s metabolites. The interaction of different abiotic factors could be used to build an integrated model. However, this question still remains unanswered: “*Is this really different from a language?”*

## Conclusion

Nowadays, the responses of plants to abiotic stress through airborne signals along with the large numbers of papers dealing with communication between plants by emitting biotic stress-induced VOCs represent the “neglected side” of plant communications. This paper emphasizes the discrepancy between the scarce information available on communication between plants subjected to abiotic stress by airborne signals and the possible ecological and practical implications for this research field. There is an urgent need to understand how plants build awareness of abiotic stress factors, especially in the era of global change and how VOCs emitted by plants subjected to abiotic stress factors interact with the environment. From an applicative point of view, an in-depth knowledge of the “VOC language” could be proficiently exploited for biomonitoring and to stimulate the biosynthesis of target metabolites (usually induced by specific abiotic stresses) to obtain clean and bioactive-enriched products. Further investigations are needed to understand the possible unwanted effect of emitter VOCs to receiver performances in plants that are subjected to abiotic stress in environmentally controlled experiments.
